# Oncologist‐patient‐caregiver decision‐making discussions in the context of advanced cancer in an Asian setting

**DOI:** 10.1111/hex.12994

**Published:** 2019-11-04

**Authors:** Chetna Malhotra, Ravindran Kanesvaran, Nesaretnam Barr Kumarakulasinghe, Sing‐Huang Tan, Ling Xiang, James A. Tulsky, Kathryn I. Pollak

**Affiliations:** ^1^ Lien Centre for Palliative Care Duke‐NUS Medical School Singapore Singapore; ^2^ National Cancer Centre Singapore Singapore Singapore; ^3^ National University Cancer Institute Singapore (NCIS) National University Health System Singapore Singapore; ^4^ OncoCare Cancer Centre Gleneagles Medical Centre Singapore Singapore; ^5^ Dana‐Farber Cancer Institute Boston MA USA; ^6^ Brigham and Women's Hospital Boston MA USA; ^7^ Cancer Control and Population Sciences Duke University Durham NC USA; ^8^ Population Health Sciences Duke University Durham NC USA

**Keywords:** cancer, decision making, patient participation, prognosis

## Abstract

**Objective:**

Patient involvement in treatment decisions is recommended in clinician‐patient encounters. Little is known about how oncologists engage patients in shared decision making in non‐Western countries. We assessed the prevalence of shared decision making among Singaporean oncologists and analysed how they discussed prognosis.

**Methods:**

We audio‐recorded 100 consultations between advanced cancer patients and their oncologists. We developed a coding system to assess oncologist encouragement of patient participation in decision making and disclosure of an explicit prognosis. We assessed patient and oncologist characteristics that predicted these behaviours.

**Results:**

Forty‐one consultations involved treatment discussions. Oncologists almost always listed more than one treatment option (90%). They also checked patient understanding (34%), discussed pros and cons (34%) and addressed uncertainty (29%). Oncologists discussed prognosis mostly qualitatively (34%) rather than explicitly (17%). They were more likely to give an explicit prognosis when patients/caregivers asked questions related to prognosis.

**Conclusion:**

Oncologists in our sample engaged their patients in decision making. They have areas in which they can improve to involve patients at a deeper level to ensure shared decision making. Findings will be used to develop an intervention targeting oncologists and patients to promote patient involvement in decision making.

## 1 INTRODUCTION

In recent decades, the patient‐physician relationship in Western countries has evolved from an asymmetrical interaction, in which patients are largely passive and physicians drive decisions, towards one where decision making is shared between patients and physicians.^1^ The concept of shared decision making has since gained considerable clinical acceptance and is recognized as the gold standard for decision making.^2^ Although current definitions vary, at its core shared decision making involves a process of making treatment decisions in a collaborative way between patients and physicians while exchanging information about different treatment options and with patients playing a major role in decisions.^2^, ^3^ This transition to shared decision making has implications particularly in discussions related to treatment options of advanced cancer.

Patients with advanced cancer often need to make tough decisions between life‐extending, burdensome and costly cancer‐directed treatments, and care that focuses primarily on preserving quality of life.^4^ When striving for patients to make informed decisions, patient‐physician discussions should involve several key elements. Physicians should provide information about patient prognosis, discuss treatment options, address pros and cons of these options and discuss the uncertainty surrounding them.^5^ Additionally, oncologists should elicit patient treatment preferences. When patients are involved in decision making, they have better outcomes, such as adherence with treatments, psychological well‐being and satisfaction with their medical care.^6^, ^7^, ^8^ Patient involvement in decision making now represents the gold standard in many Western countries.^1^, ^6^

In most Asian countries, however, the patient‐physician relationship is still largely paternalistic. Singapore (the setting of this study), a multicultural South‐East Asian country with a developed economy, is currently undergoing the transition from a paternalistic to a shared decision‐making model.^9^ Studies show that many Singaporeans desire complete information regarding their illness and prefer to be involved in making treating decisions with their physician.^10^, ^11^ As a result, there is a growing need for Singapore physicians to not just be technical experts but also to encourage patients and caregivers to be active participants in decision making. Within the context of advanced cancer consultations, physicians will also need to have an explicit discussion of prognosis without which patients may overestimate their prognosis and chance of cure and be unable to make truly informed decisions.^12^, ^13^, ^14^ Further, in Singapore and in many other countries, family caregivers have greater power or control over patient's treatment decisions, particularly if the patient is older or less educated.^15^ There is anecdotal information to suggest that such patients may be excluded from the decision‐making process. However, currently there is sparse literature from Asian countries describing the extent of physician encouragement of patient participation in decision making, explicit prognostic discussions and involvement of caregiver only during consultations. Most studies rely on patient perceptions of their involvement in decision making and reports of their prognostic understanding, acknowledging that perceptions may be systematically biased by recall, low expectations of the patient themselves to be involved in decision making and their hope of a cure.^16^, ^17^ Few have looked at actual encounters.

The aim of this study was to describe the prevalence of shared decision making among Singaporean oncologists and how oncologists discussed prognosis. Our long‐term goal involves designing efforts to improve patient involvement in decision making in this Asian setting.

## 2 METHODS

### 2.1 Sample

Between March 2015 and January 2016, the study team first approached medical oncologists from two large cancer centres in Singapore and obtained their written consent to participate in the study. These two centres see more than 70% of all cancer patients in Singapore.^18^ Subsequently, we screened eligible patients of participating oncologists from medical records and approached them in waiting rooms of outpatient consultation clinics to obtain their written informed consent. Patient inclusion criteria were having diagnosis of a stage IV cancer and holding Singapore permanent residence or citizenship. Patients under 21 years and those cognitively impaired require consent from their legal guardian/parent to take part in research and were excluded. Cognitive impairment was assessed based on documentation in medical records. Primary informal caregiver included those most involved in providing care to the patient or ensuring provision of care or in making decisions regarding the patient's treatment or care. We excluded paid domestic helpers. The study was approved by the SingHealth Institutional Review Board.

### 2.2 Surveys

We placed a digital audio recorder unobtrusively in the consultation room to audio‐record oncologist‐patient consultations. We audio‐recorded consultations rather than video recording or directly observing because audio recording is unobtrusive, less likely to modify naturally occurring behaviours during consultation, allows repeated reviewing of data for several behaviours and by multiple study team members and is less expensive.^19^ Oncologists were not aware of the outcomes being assessed in the study. The consultations included both new cases and follow‐up cases. Each patient/caregiver was recorded only once. We surveyed oncologists and collected data on their age, gender and clinical grade. We also surveyed patients and caregivers pre‐consultation about their demographics (age, gender, ethnicity, marital status and education).

### 2.3 Analysis

This study involved applying a codebook (Appendix S1) to create quantitative counts for the elements of shared decision making. We translated (when necessary) and transcribed verbatim all audio recordings to allow a detailed study of consultations. Native language speakers checked transcriptions and translations extensively for quality. Two coders coded together all consultations.

We first identified the treatment decision being made during the consultation. We counted and recorded the number and type of treatment decisions being made in each consultation. These included starting or not starting a treatment, continuing or stopping treatment, starting another round of treatment immediately or after waiting for some time, and choosing between treatment options. Within the context of each treatment decision, we developed a coding system to assess three outcomes: (1) oncologist encouragement of patient participation in decision making, (2) oncologist likely involvement of caregiver only in decision making and (3) oncologist disclosure of prognosis. Although our coding system was based on a review of physician behaviours included in three existing coding systems for shared decision making (Observing Patient Involvement in Decision Making (OPTION), Decision Support Analysis Tool and Decision Analysis System for Oncology), it was not derived from any one system in particular.^20^ We chose not to use any of these existing tools as their validity has not been evaluated in our local setting. Some aspects of decision making unique to our local context such as oncologist involvement of only the caregiver in pertinent aspects of decision‐making conversations (outcome 2) are not included in existing tools. Further, two of three existing tools have not specifically been developed for use with advanced cancer patients and thus do not include an assessment of prognosis discussions (outcome 3) which is essential to allow patients to make informed decisions regarding their treatment.

For the first outcome, we coded five oncologist behaviours signalling their encouragement of patient participation in treatment discussions. These included (a) oncologist checked patient perception or understanding of test results, treatment options and prognosis (either before or after disclosure), (b) oncologist listed more than one treatment option, (c) oncologist discussed pros and cons of each treatment option (at least one of each was discussed), (d) oncologist discussed treatment uncertainty with each option (letting the patient know that it is unclear whether the treatment will work) and (e) oncologist checked patient preference or deferred to patient preference. We coded each behaviour when it was present in the consultation. We scored each consultation based on the number of behaviours exhibited. Total score ranged from 1 to 5.

For the second outcome, we identified oncologist behaviours that may have affected patient participation in decision making by possibly excluding the patient from the conversation. This included two types of behaviours—(a) oncologist, when discussing treatment pertinent information, switched language from patient's primary language (eg Mandarin) to English and (b) oncologist discussed treatment pertinent information with only the caregiver after the caregiver asked the patient to step out of the consultation room. We scored each consultation as 0 or 1 based on the presence or absence of any of these two behaviours.

Lastly, we coded how prognostic information was communicated, for instance in either numerical terms (eg as ‘life expectancy’ and ‘median survival’); or semi‐quantitative terms (eg ‘a few more weeks/months’, ‘months not years’); or qualitative terms (eg ‘not much time left’, only saying that ‘the condition was incurable’ without giving any specific information on survival, or just saying that ‘the disease was advanced’ or ‘looked bad’ but not giving any further information). We coded each instance of prognostic discussion as 0 (=no prognosis disclosed), 1 (=qualitative prognosis) or 2 (=numerical or semi‐quantitative prognosis). We then scored each consultation based on the highest code assigned within that consultation.

We used a Poisson regression model predicting the number of oncologist behaviours signalling their encouragement of patient participation in treatment discussions. To assess whether oncologist behaviours varied by key patient and oncologist characteristics, we included as independent variables patient age, gender, marital status and education as well as oncologist age, gender, clinical grade and country of basic medical training and consultation length.

We used chi‐squared test to assess the association of oncologist involvement of caregiver only with patient (age, gender, education, ethnicity and marital status), caregiver (age, gender, education, ethnicity and marital status) and oncologist characteristics (age, gender and clinical grade).

Finally, using an ordered logistic regression model, we assessed the association between oncologist disclosure of a more explicit prognosis (0 = no prognosis disclosed, 1 = qualitative prognosis, 2 = numerical or semi‐quantitative prognosis). Again, to test variation in oncologist disclosure of prognosis by important patient and oncologist characteristics, the following independent variables were included—patient age, gender, ethnicity, marital status, education and whether patient asked prognosis‐related question, and oncologist age, gender, clinical grade and country of basic medical training. 

We used NVivo for all coding and Stata for all analyses.

## 3 RESULTS

### 3.1 Sample characteristics

We approached 43 oncologists to participate in the study, and 37 (86%) agreed. Of these, we audio‐recorded consultations of 30 oncologists. Out of the 230 eligible patients approached, 113 (49%) patients and their primary informal caregivers consented to participate. A total of 100 out of 113 patients who consented (88%) completed a pre‐consultation survey and had their consultations audio‐recorded. Of these 100, 77 had a primary informal caregiver accompanying them who also consented and completed the surveys.

We excluded 59 consultations from analysis because they did not contain any decision making—19 did not involve any treatment discussions and 40 only involved brief discussions about continuing treatment as scheduled and there was no dialog about it. The remaining 41 consultations involved either new treatment options or changes to the current treatment plan. Nineteen (46%) involved discussions surrounding only one aspect of treatment choice (eg choosing between treatment types such as chemotherapy, surgery or radiotherapy) and 22 (54%) involved discussions surrounding two or more aspects of treatment choice (eg between treatment types and time of treatment initiation, such as initiating treatment now or waiting for a few months). Stopping active life‐prolonging treatments and referral to palliative/hospice care was discussed as an option in 5 (12%) of the consultations.

There were no significant differences in the characteristics of patients and caregivers between the analytic sample of 41 consultations and the overall sample. In the analytic sample of 41 consultations, patients were on average 60.6 (SD = 12.7) years of age, mostly females (56%), Chinese (73%) and married (83%). 15% of the patients had not received any formal education. Caregivers were on average 49.4 (SD = 15.4) years of age, mostly female (66%), Chinese (71%) and married (86%). Caregivers were, on average, better educated than patients, with 31% having university education and above (Table 1).

**Table 1:** Demographics of patients, caregivers and oncologists

Demographics	Overall sample (n = 100)		Analytic Sample (n = 41)		Oncologists (n = 25) n (%)
	Patients (n = 100)	Caregivers (n = 77) n (%)	Patients (n = 41) n (%)	Caregivers (n = 35) n (%)	
Age, mean (SD)	59.8 (12. 9)	50.1 (15.8)	60.6 (12.7)	49.4 (15.4)	40.0 (7.9)
Gender (male)	49	33 (42.9%)	18 (43.9%)	12 (34.3%)	16 (64.0%)
Ethnicity					
Chinese	75	57 (74.0%)	30 (73.2%)	25 (71.4%)	20 (80.0%)
Malay	15	11 (14.3%)	7 (17.1%)	6 (17.1%)	1 (4.0%)
Indian	4	5 (6.5%)	1 (2.4%)	2 (5.7%)	4 (16.0%)
Other	6	4 (5.2%)	3 (7.3%)	2 (5.7%)	0
Marital status					
Married	77	58 (75.3%)	34 (82.9%)	30 (85.7%)	—
Not married	23	19 (24.7%)	7 (17.1%)	5 (14.3%)	—
Educational status					
No formal education	11	2 (2.6%)	6 (14.6%)	1 (2.9%)	—
Primary	21	12 (15.6%)	6 (14.6%)	4 (11.4%)	—
Secondary	38	20 (26.0%)	15 (36.6%)	7 (20.0%)	—
Vocational/ITE	3	2 (2.6%)	2 (4.9%)	2 (5.7%)	—
Junior college/Polytechnic/Diploma	11	16 (20.8%)	3 (7.3%)	10 (28.6%)	—
University and above	14	24 (31.2%)	7 (17.1%)	11 (31.4%)	—
Don't know/Can't remember	2	1	2 (4.9%)	0	—
Clinical grade					
Senior resident/fellow	—	—			7 (28.0%)
Associate consultants/consultants/senior consultants	—	—			18 (72.0%)

Twenty‐five oncologists were involved in the 41 consultations analysed. They were, on average, 40 (SD = 7.9) years old, 72% were male and 28% were senior residents/fellows (Table 1). In all consultations, the oncologist spoke the same language as the patient and/or caregiver.

### 3.2 Oncologist encouragement of patient participation in decision making

Table 2 shows the extent to which oncologists showed each of the five behaviours encouraging patient participation in decision making. In most consultations, oncologists listed more than one treatment option (90%). They also checked patient's understanding (34%) and discussed pros and cons (34%), and uncertainty (29%) for all treatment options. Oncologists showed at least one of these behaviours in all consultations and all of these behaviours in only 2 (5%) consultations. On average, oncologists exhibited 2.7 (SD = 1.1) of these five behaviours in each consultation. Oncologists showed more of these five behaviours when patients were older than 60 years old (*β*: 0.25; *P* = .02). The number of behaviours did not vary by other patient or oncologist characteristics (Table 3).

**Table 2:** Oncologist encouragement of patient participation in decision making, their likely involvement of only the caregiver and discussion of prognosis (n = 41)

Codes	Number of encounters n (%)	Illustrative quotes
*Oncologist encouragement of patient participation in decision making*		
Oncologist checked patient understanding	14 (34.2%)	*‘Why don't you share with me what the surgeons have told you so far? Could you understand what your condition, so far?’*
Oncologist listed more than one treatment option	37 (90.2%)	*‘In terms of the treatment wise ah, options are still the same, ok? Chemotherapy is one option.. Second option is the one that I told you, the radio frequency ablation. Third option like what I've discussed with you at the time, surgery, ok?’*
Oncologist discussed pros and cons of all treatment options	14 (34.2%)	*‘It's not chemo… so it shouldn't make you weak or that kind of thing, you know… vomiting… drop hair… shouldn't cause that kind of things. But it still got side effects. So it can cause… inflammation inside the lung, some people get inflammation inside the… liver… inflammation inside the… intestine… also can get diarrhea, they can get liver pain… can get breathless and things like that la. Those are… uncommon side effects. The probability about 1% to 3% la’.*
Oncologist discussed uncertainty of all treatment options	12 (29.3%)	*‘We have oral chemo…very mild. Mild means err…not much of side effect…but some people respond to oral chemo alone. Maybe 30% of patients, when we give oral chemo, they shrink. The other 70%, no response. If we combine oral chemo plus an injection…okay, maybe 40%, 50%…more people will respond. The other 60%, will still not respond’.*
Oncologist checked patient preference or encouraged patient to ask questions or deferred to patient recommendation	32 (78.1%)	*‘Probably… what I want to do is, actually, to let you have the information to… to consider first. Maybe you can decide… maybe, within these few weeks or… ya… then… consider la…’*
*Oncologist likely involvement of caregiver only*	6 (14.6%)	*‘[Doctor to the caregiver of a Hokkien speaking patient] Ya, from the whole long process la, ho? Having said that, her condition definitely has deteriorated quite a lot since the last time I saw her. Ok? So although we have, she…she had, we have maintained her…her…how so called how to say, maintain her…her fitness, her activity levels up to some point, I think we are seeing quite a fast decline at this moment la’*
*Discussion of prognosis*	21 (51.2%)	
Explicit prognosis (numerical or semi‐quantitative)	7 (17.1%)	*‘Across the board for stage 4 patients, the…the average survival we are looking at is anywhere from one and a half, to two and a half years’.*
Qualitative prognosis	14 (34.2%)	*‘To cure this cancer, not possible lah, not possible’.*

**Table 3:** Patient and oncologist predictors of number of oncologist behaviours encouraging patient participation in decision making and oncologists' disclosure of explicit prognosis

	Number of oncologist behaviours encouraging patient participation in decision making^a^ (N = 38)			Disclosure of a more explicit prognosis^b^ (0 = no prognosis disclosed, 1 = qualitative prognosis, 2 = explicit prognosis (numerical or semi‐quantitative) (N = 38)		
	Coeff.	*P*	95% CI	OR	*P*	95% CI
Patient age ≥60 y (Ref: age <60 y)	0.30	.01	0.06 to 0.54	7.47	.01	1.49‐37.3
Patient gender: Female (Ref: Male)	0.21	.12	−0.05 to 0.48	1.32	.73	0.27‐6.56
Patient ethnicity: Malay/Indian/Other (Ref: Chinese)	−0.06	.67	−0.35 to 0.23	1.36	.74	0.22‐8.57
Patient marital status: Separated/Widowed/Divorced/Never married (Ref: Married)	−0.02	.92	−0.42 to 0.38	1.34	.80	0.14‐12.57
Patient educational status: Secondary or above (Ref: Primary or below)	0.01	.95	−0.25 to 0.26	2.23	.54	0.17‐28.51
Patient/Caregiver asked questions about prognosis (Ref: did not ask about prognosis)	—	—	—	50.59	<.01	6.24‐410.17
Oncologist age	−0.01	.29	−0.04 to 0.01	0.91	.21	0.79‐1.05
Oncologist gender: Female (Ref: Male)	0.13	.31	−0.17 to 0.37	0.22	.15	0.03‐1.75
Oncologist clinical grade: Associate Consultant/Consultant/Senior Consultant (Ref: Senior resident/fellow)	0.30	.11	−0.06 to 0.67	0.93	.97	0.03‐28.19
Oncologist country of basic medical training: Other countries (Ref: Singapore)	0.12	.45	−0.20 to 0.44	0.82	.79	0.20‐3.44
Consultation length	0.01	.002	0.004‐0.01	—	—	—

a Using a Poisson regression model.

b Using an ordered logistic regression model.

### 3.3 Oncologist involvement of caregivers only in decision making

In 6 (15%) consultations, oncologists discussed pertinent information about the patient's treatment only with the caregiver in a way that may have excluded the patient from decision making. Of these consultations, in 4 (10%) consultations, they spoke to the patient in one language and in another language with the caregiver. When discussing vital information such as scan results and treatment options, they switched from the patient's preferred language to another language (eg from Mandarin to English). It was unclear whether the patient understood the language spoken to the caregiver. In 2 (5%) consultations, the oncologist discussed prognosis with only the caregiver after the patient was asked to leave the consultation room by the caregiver.

Oncologists were more likely to involve only the caregiver in consultations with patients older than 60 years (24% vs 0%, *P* < .05) and when patients were lower educated (25% vs 4%, *P* < .05). This behaviour did not vary significantly by any other patient, caregiver or oncologist characteristics.

### 3.4 Oncologist disclosure of prognosis

Oncologists discussed prognosis in 21 (51%) consultations. They provided a more explicit prognosis to only 7 (17%) of the patients, which included a numerical prognosis to 6 (15%) patients and a semi‐quantitative prognosis to only 1 (2%) patient. They gave a qualitative prognosis to 14 (34%) of the patients which included telling 8 (20%) patients that their disease was incurable, 3 (7%) that it was advanced, 1 (2%) that it looked bad and 2 (5%) that they had been cured (Table 2). Only in 8 (20%) consultation, patients/caregivers asked oncologist questions related to their prognosis.

Table 3 shows that oncologists were more likely to provide a more explicit prognosis to patients older than 60 years (OR: 7.5; *P* < .05) and to those patients/caregivers who asked questions related to prognosis (OR: 50.6; *P* < .01). There was no variation in providing prognostic information by other patient and oncologist demographics.

## 4 DISCUSSION

In a multi‐ethnic Asian setting, we analysed consultations between oncologists and patients with stage IV cancer, most of whom were accompanied by their caregivers. First, we found that oncologists displayed some behaviours that encourage decision making. We also found that in some areas, their behaviours could have been more frequent. Second, there were instances in which the oncologists discussed pertinent information only with the caregiver in a way that may have excluded the patient from decision making; this behaviour was also more likely to occur with older patients. Third, they mostly disclosed prognosis less explicitly and were more likely to give an explicit prognosis when patients or caregivers asked questions related to their prognosis.

We found that as recommended, oncologists listed more than one treatment option in most consultations. Oncologists also did things to engage patients but to a lesser degree. For instance, they checked patient understanding and discussed both pros and cons and uncertainty related to all treatment options. They did this in about one‐third of the encounters, which is impressive given that anecdotal information suggests that these oncologists have not received any extensive training in shared decision‐making communication beyond a few lectures and role plays in medical school. Providing information on both pros and cons may help patients to better weigh their treatment options to make an informed choice. Our results corroborate previous findings from many countries that discussion of pros and cons may not always happen during consultations.^21^, ^22^, ^23^, ^24^, ^25^, ^26^ Studies also suggest that even when such discussion happens, they may be incomplete.^27^ Especially in the context of advanced cancer, treatment options often have uncertain benefits and decisions need to be made by weighing the pros and cons and the uncertainty related to each option. Such discussions are therefore an essential element of shared decision making. Oncologists may benefit from a specialized communication training to conduct such discussions.^28^, ^29^ A possible barrier to such discussions may be the short consultation time as also suggested by our study findings. Average consultation time for visits recorded in our study was 15 minutes compared to 23 minutes in the United States.^30^

Oncologist involvement of only the caregiver in pertinent aspects of decision‐making conversations, though relatively rare, continues to exist. In Singapore and in many other Asian countries, family caregivers are known to have more power or control over patient's treatment decisions, particularly if the patient is older or less educated.^15^ During decision making, family involvement has been seen as playing a key role as compared to the emphasis on patient autonomy in most Western countries.^15^, ^31^, ^32^ Consistent with Confucian principles of decision making, family members feel the responsibility to make decisions for the patient. In this regard, oncologist can choose not to disclose diagnosis and/or prognosis to maintain patient's hope and in keeping with the best interest of the patient.^32^, ^33^ However, as policy makers in Singapore^9^ and many other countries call for a shared decision‐making model, oncologists may need to ensure that all patients, especially those who are older, have the opportunity to participate in the decision‐making process should the patient wishes to do so. They can elicit patient preferences for information and decision making, and respond to family concerns regarding conveying prognosis and other treatment‐related information directly to patients. If patients prefer that their families be given information rather than them, then it should be ethically acceptable to do so.

It was encouraging that when patients and caregivers specifically asked questions about their prognosis, oncologists responded explicitly. Several studies have shown that patients want to be told about their prognosis in a sensitive but honest manner^34^, ^35^; this has also been found in several Asian countries such as Taiwan, Japan, Nepal and Hong Kong.^36^, ^37^, ^38^, ^39^, ^40^ Oncologists may, in general, withhold explicit prognostic information from patients due to fear of making patients emotionally overwhelmed.^41^, ^42^, ^43^, ^44^ Younger patients less than 60 years have been seen to be more worried of cancer diagnosis and poor prognosis^45^, ^46^, ^47^, ^48^ possibly making oncologists in our study less likely to disclose their prognosis. Explicit discussions about prognosis may be necessary for patients to make informed decisions. In the absence of explicit prognostic information, many patients report not knowing or misunderstanding the status of their disease and intent of treatment.^49^ For instance, a systematic review reported that up to 75% of the patients are unaware of their poor prognosis.^50^ A study from the United States found that 69% of patients with advanced lung cancer and 81% of patients with colorectal cancer did not believe that their chemotherapy was not curative though the odds for more inaccurate beliefs were higher among patients who rated their communication more favourably.^13^ Training oncologists to convey prognosis by first asking permission from patients to do and then conveying that information in a sensitive manner as well as empowering patients to actively ask questions related to their prognosis may increase the frequency of explicit prognostic discussions during consultations.

The main limitation of this study is the small sample size and that a large number of consultations were excluded as they did not involve any treatment discussion. We were also unable to differentiate consultations based on whether these were new or follow‐ups and based on length of relationship between oncologist and patient. Second, we do not know the extent to which patient wished to be involved in the decision‐making process. Third, since this was a cross‐sectional study design, we have no knowledge about additional information communicated to patients or on the oncologist behaviour during other visits with the same patients. Fourth, we are unable to comment on whether all relevant treatment options were presented and whether all possible pros and cons including significant side‐effects were discussed in a balanced way during the consultation. We are also unable to distinguish to what extent oncologists' listing of treatment options gave patients the appearance of choice vs a substantive choice. Fifth, for the 4 consultations in which oncologist switched from patient's preferred language to another language while discussing treatment information, we did not explicitly ask patients whether or not they were able to understand what was being spoken in the other language. Lastly, inter‐rater agreement could not be calculated as coding was done together by the two coders instead of separately. The strength of this study is that it adds to the sparse literature quantifying oncologists' encouragement of patient participation in decision making and providing prognostic information in an Asian setting.

In conclusion, this study shows that as recommended, Singaporean oncologists almost always listed more than one treatment option in most consultations. They also displayed other behaviours that encourage patient involvement. They potentially could do these at a higher level with some training. We hope to use these findings to develop interventions for both oncologists and patients to promote provision of information by oncologists and encourage effective patient participation and autonomy in decision making.

## CONFLICT OF INTEREST

None.

## AUTHOR CONTRIBUTIONS

CM was the principal investigator of the study. CM, RK, JAT and KIP were involved in conception and study design. CM, RK, NBK, ST and KP were involved in the collection and assembly of the data. CM, RK and KIP were responsible for data analysis and interpretation. All authors contributed to the preparation and writing of the manuscript and approved the final manuscript.

## INFORMED CONSENT

I confirm all participant identifiers have been removed or disguised so the participants described are not identifiable and cannot be identified through the details of the story.

## Data Availability

The data that support the findings of this study are available on request from the corresponding author. The data are not publicly available due to ethical restrictions.
