# Supplementary material for: Oncologist‐patient‐caregiver decision‐making discussions in the context of advanced cancer in an Asian setting
Source: Health Expect. 2019 Nov 4;23(1):220–8. doi: 10.1111/hex.12994 (PMC6978867; doi:10.1111/hex.12994)
Supplement: Supplementary file 1 [file HEX-23-220-s001.docx]

**Codebook for Treatment Decision-making:** Code conversations between physician and patient only

*Use only when specific treatment decisions are being made. If no treatment decision is being made, then the codes will not apply.*

***First, identify the treatment decision being made****. In one transcript, there may be more than 1 treatment decisions being made. In the end,* ***count and record the number and type of treatment decisions being made in each transcript.***

Treatment discussion examples: Treatment/No treatment, Start treatment/Wait, Treatment options: Chemo/Surgery/Radiation/Clinical trial…, Chemo: Medicine A/Medicine B…

***Use the following codes for each treatment decision being made****.*

1. **Oncologist encouragement of patient participation in decision-making**
2. **Physician checks patient perception/understanding (before and after disclosing information):** Ask if patient knows test results, treatment options, prognosis, and other cancer-related information
3. **Physician lists more than one treatment option**

[Code only when all options are mentioned]

1. **Physician discusses pros and cons of each treatment option with patient** [Code only when pros and cons are discussed for each treatment option]
2. **Physician discusses treatment uncertainty with each option:** Letting the patient know that it is unclear whether it is going to work

[Code only when uncertainty is discussed for each treatment option]

1. **Physician checks patient preference, encourages patient to ask questions, defers to patient recommendation**
2. **Oncologist likely involvement of caregiver only in decision-making**
3. **Physician involves only caregiver not patient:** Switches language to talk to caregiver, leaves patient out of the conversation
4. **Oncologist disclosure of prognosis**
5. **Physician** **discusses prognosis/goals**: Physician acknowledges disease severity, conveys risk, discuss goals of cure, whether or not curative

Indicate how information about survival benefit is communicated (choose category based on the maximum information provided) – **(4a)** **numerical prognosis** (e.g. about 4 weeks), **(4b)** **semi-quantitative** **prognosis** (e.g. a few more weeks/months, months not years), **(4c)** **qualitative prognosis** (e.g. not much time left), **not curable**, no specific information on survival given, **advanced/stage IV**/ severe, no information on prognosis or whether or not it is curable, **bad/not good,** no information on prognosis or whether or not it is curable or advanced).

**Notes:**

- Do NOT code scheduling test/treatment
